# Sildenafil Enhances the Therapeutic Effect of Islet Transplantation for Diabetic Peripheral Neuropathy via mTOR/S6K1 Pathway

**DOI:** 10.1155/2023/8199029

**Published:** 2023-10-07

**Authors:** Xiandong Zhu, Shangjing Xie, Jiawei Chen, Qiaohong Lu, Xiaowu Wang, Feixiang Duan, Sinian Xu, Yan Zhang, Hongjian Huang, Yongqiang Wang, Hongwei Wang, Bicheng Chen, Huanjie Huang

**Affiliations:** ^1^Department of Thyroid Surgery, The First Affiliated Hospital of Wenzhou Medical University, Wenzhou 325000, Zhejiang Province, China; ^2^Key Laboratory of Diagnosis and Treatment of Severe Hepato-Pancreatic Diseases of Zhejiang Province, The First Affiliated Hospital of Wenzhou Medical University, Wenzhou 325000, Zhejiang Province, China; ^3^Department of Neurosurgery, Wenzhou Central Hospital, Affiliated Dingli Clinical Institute of Wenzhou Medical University, Wenzhou 325000, Zhejiang Province, China; ^4^Department of Neurology, The First Affiliated Hospital of Wenzhou Medical University, Wenzhou 325000, Zhejiang Province, China

## Abstract

**Purpose:**

This study aimed to investigate the potential mechanism underlying the therapeutic effect of sildenafil in combination with islet transplantation for diabetic peripheral neuropathy.

**Methods:**

A streptozotocin-induced diabetic mouse model was established to evaluate the effects of islet transplantation and sildenafil intervention. The mice were subjected to different interventions for 6 weeks, and histopathological staining and immunohistochemistry techniques were employed to examine the pathological changes and protein expressions of BDNF, MBP, and cleaved caspase-3 in the sciatic nerve tissue. Moreover, RSC96 cells were cocultured with islet cells and sildenafil under high glucose conditions to investigate the potential involvement of the mTOR/S6K1 pathway, BDNF, and MBP proteins. Western blotting was used to detect protein expression in each group.

**Results:**

The results showed that islet transplantation can restore sciatic nerve injury in diabetic mice, and sildenafil can enhance the therapeutic effect of islet transplantation. In addition, the combination of sildenafil and islet cells significantly upregulated the expression levels of mTOR/S6K1, BDNF, and MBP in RSC96 cells under high glucose conditions.

**Conclusions:**

Islet transplantation can reverse sciatic nerve injury in diabetic mice, and islet cells exhibit a protective effect on RSC96 cells under high glucose conditions via the activation of the mTOR/S6K1 pathway. Sildenafil enhances the therapeutic effect of islet transplantation, which may represent a potential treatment strategy for diabetic peripheral neuropathy.

## 1. Introduction

Diabetic peripheral neuropathy (DPN) is one of the most common complications among individuals diagnosed with type I or type II diabetes [1]. DPN is characterized by distal symmetrical polyneuropathy and autonomic neuropathy. Approximately 50% of diabetic patients develop neuropathy, often without awareness of its onset due to its gradual progression. The prognosis for DPN patients is often poor and includes a heightened risk of amputation, posing physical and psychological burdens on patients [2, 3]. One of the key components of myelinated nerve fibers is the myelin sheath structure composed of Schwann cells, which plays an important role in maintaining the normal structure and morphology of nerve fibers, nutritive nerve [4], and nerve injury repair and regeneration [5]. Schwann cell injury is a primary pathological characteristic of DPN [6]. In diabetic rat sciatic nerve tissue, nerve axons were irregular and atrophied, while the myelin sheath was uneven in thickness and damaged [7]. Some studies have found that under the condition of high glucose, the apoptosis of rat Schwann cells increased and the expression of brain-derived neurotrophic factor (BDNF) decreased [8]. BDNF is implicated in the expression of myelin basic protein (MBP) [9], which is essential for maintaining the normal physiological function and structure of the myelin sheath. Therefore, it is worth investigating approaches for reducing and repairing Schwann cell damage as a potential avenue for treating DPN.

Islet transplantation was reported to be an effective *β*-cell replacement therapy because of its good effect in stabilizing blood glucose [10]. With the optimization and development of islet isolation, islet transplantation has increasingly become a practical and feasible treatment for patients with type I diabetes. A study of 84 patients who underwent islet transplantation revealed that 23 (68%) received single islet transplants, while 47 (94%) received two transplants and maintained islet activity for 12 months. In 64% of patients who received one or two times of transplant, islet function remained normal for around 6 years [11]. Multiple studies have corroborated the effectiveness of islet transplantation in mitigating not only diabetes itself but also diabetic complications, including diabetic nephropathy [12], diabetic cardiomyopathy [13], and diabetic testicular injury [14]. However, we note that there are few studies that delve into the specific function and mechanism of islet transplantation in diabetic peripheral nerves.

Sildenafil, a classic phosphodiesterase-5 inhibitor (PDE5 inhibitor), is commonly used as a clinical drug for the treatment of male erectile dysfunction [15]. Recent studies have also explored the potential benefits of sildenafil for the treatment of pancreatitis due to its anti-inflammatory, antioxidative, and antiapoptotic properties [16]. Clinical cases have reported that some patients with both erectile dysfunction and diabetic peripheral neuropathy (DPN) experienced relief of DPN symptoms after taking sildenafil [17]. Experimental studies have further demonstrated that PDE5 inhibitor treatment may enhance vascular function and axon remodeling, ultimately improving the neurological function of diabetic mice with peripheral neuropathy [18]. Furthermore, previous studies have demonstrated that sildenafil can provide protection to rat adrenal pheochromocytoma cell PC12 under high glucose conditions [19]. In addition, sildenafil has also been found to activate mitochondrial ATP-sensitive potassium channels, which could potentially confer resistance to tissue ischemia injury [20]. Moreover, a recent study suggested a possible immunosuppressive effect of sildenafil *in vitro*. Therefore, we aim to investigate the potential effects of sildenafil on DPN and explore the possibility of combining sildenafil with islet transplantation as a novel therapeutic approach for DPN.

Mammalian target of rapamycin (mTOR) is a Ser/Thr kinase, which participates in a number of physiological and pathological processes, such as growth, signal integration, cancer, diabetes, and aging [21]. mTOR is a double-edged sword for diabetes. In different cells, it can promote and restrict diabetes. Studies have found that inhibition of mTOR can protect renal function and reduce apoptosis in diabetic mice [22], while activation of mTOR can restore cardiac injury in diabetic mice [23]. Ribosomal protein S6 kinase 1 (S6K1) is the main downstream target of mTOR and is a member of the AGC kinase family that has been extensively studied in various metabolic, inflammatory, aging-related, and nervous system diseases and cancers [24]. During the differentiation of Schwann cells from amniotic fluid stem cells, S6K1 regulates nerve growth factor receptor (NGFR), glial fibrillary acidic protein (GFAP), central nervous system specific protein S100 *β*, and other related proteins [25]. Consequently, S6K1 is a promising target for the development of treatments to alleviate peripheral nervous system damage. This study aims to investigate changes in these proteins in diabetic peripheral neuropathy (DPN).

The objective of this investigation is to evaluate the efficacy of islet transplantation and sildenafil on diabetic peripheral neuropathy (DPN) both *in vivo* and *in vitro* and to explore the potential of combining these treatments as a therapy for DPN. Our hypothesis is that active islets can activate the mTOR/S6K pathway, leading to the restoration of BDNF and MBP expression in Schwann cells, ultimately resulting in the mitigation of peripheral nerve injury. Furthermore, we postulate that the inclusion of sildenafil can enhance the activity of transplanted islets, shield them from damage, and potentiate their function.

## 2. Materials and Methods

### 2.1. Cell Culture Conditions

The rat Schwann cell line RSC96 (iCell Bioscience Inc., Shanghai) was cultured in DMEM medium with 10% fetal bovine serum, 100 U/ml penicillin, and 100 U/ml streptomycin and the cell incubator was routinely maintained at 37°C and 5% CO_2_. For treatment condition, RSC96 was cultured in 6-well plates and divided into 7 different groups, which were 5 mM of D-glucose in DMEM as the control group (C), 5 mM of D-glucose as the DMEM + 95 mM mannitol group (mannitol), 100 mM D-glucose as the high glucose group (HG), 100 mM D-glucose + 0.1 *μ*M sildenafil (HG + sildenafil), 100 mM D-glucose + islets (HG + I), 100 mM D-glucose + 0.1 *μ*M sildenafil + islets (HG + I + sildenafil), 100 mM D-glucose + islets + 0.1 *μ*M rapamycin (HG + I + rapamycin), and 100 mM D-glucose + 0.1 *μ*M sildenafil + islets + 0.1 *μ*M rapamycin (HG + I + sildenafil + rapamycin). All groups were cultured for 48 h.

### 2.2. Animal Experiments

Mature male C57BL/6 mice (8-week-old, 20–25 g) were purchased from the Laboratory Animal Center (Wenzhou Medical University, Wenzhou, China). The environment mice accommodated in were specified pathogen free (SPF) at 24°C ± 1°C and on a 12-hour light/dark cycle for at least one week before the experiment. Then, twenty mice, which fasted for 6–8 hours before every injection, were injected with streptozotocin (STZ, 60 mg/kg, Sigma Aldrich, St. Louis, USA) intraperitoneally for 5 consecutive days to induce diabetes (blood glucose > 16.67 mmol/L).

After a 16-week feeding, the diabetic mice were randomly divided into 4 groups: DB group (*n* = 5, mice with diabetes), DB + sildenafil group (*n* = 5, DB mice intragastric administration of sildenafil, 20 mg/kg per day, Pfizer Inc., York, USA), DB + IT group (*n* = 5, DB mice treated with islet transplantation), and DB + IT + sildenafil group (*n* = 5, DB mice treated with islet transplantation and sildenafil). There were another five healthy mice were marked as the control group (control). All animals were sacrificed and the tissues were collected 4 weeks after different treatments.

### 2.3. Islet Transplantation

Mature male C57BL/6 mice used as islet donors were anesthetized with isoflurane. After ligation of the ampulla of Vater, 5 mL collagenase V (Gibco, CA, USA) was injected into the pancreatic duct via the common bile duct. The swollen pancreas was then removed and put into a 37°C waterbath for digestion. Islets were isolated by density gradient centrifugation with Histopque-1077 and picked up into a black glass petri dish. Then, they were cultured in RPMI-1640 (Gibco, California, USA) for 4–6 hours, and the viability of the islets was evaluated by FDA-PI (fluorescein diacetate-propidium iodide, Solarbio, Beijing, China) before surgery. After exposing the diabetes mouse's kidneys, islets equivalent to 250–350 islets (IEQ) were slowly injected into the renal capsule.

### 2.4. Hematoxylin-Eosin (HE) and Luxol Fast Blue Staining

After being fixed with 4% paraformaldehyde, the renal and sciatic nerve tissues were cut into 5 *μ*m paraffin samples. For HE staining, the sections were performed as previously described [14]. For Luxol fast blue staining, the sciatic nerve samples were then deparaffinized and hydrated in 95% ethanol. They were put into Luxol fast blue solution (Solarbio, Beijing, China) for staining overnight at room temperature. 95% ethanol was used to wash away excess dyes. The sections were put into lithium carbonate and 70% ethanol, respectively. After dehydration, clearing, and mounting, the pathological changes of sciatic nerves were observed under a light microscope, and the diameter of axons and nerve fibers and the thickness of myelin sheath were measured.

### 2.5. Immunohistochemistry

The paraffin renal and sciatic nerve tissues were dewaxed in xylene for 2 hours. After gradient dehydration in alcohol, the samples were boiled for 15 minutes with maximum power in citric acid-hydrochloric acid solution (0.01 mol/L). The sections were incubated in hydrogen peroxide (3% H_2_O_2_) for 10 minutes to reduce the effect of endogenous peroxidase and then blocked with 5% normal goat serum. After an overnight incubation with primary antibodies against insulin (Cell signal Technology Company, Boston, USA), BDNF (Affinity, USA), MBP (Proteintech, USA), and cleaved caspase-3 (Cell signal Technology Company, Boston, USA) at 4°C, the sections were further covered with peroxidase-coupled secondary antibody for 1 hour. At last, sections were stained with diaminobenzidine (Beyotime, China). Positive areas were measured by calculating the mean integrated optical density (IOD).

### 2.6. Western Blotting

The concentration of total cell protein was measured by BCA protein detection kit (Beyotime, China, Jiangsu). Then, the proteins were dispersed by size via 10% SDS-PAGE, and the resultant bands were transferred to PVDF membranes. After being blocked with 5% skimmed milk powder supplemented with TBST buffer, the membranes were incubated with primary antibodies at 4°C overnight. The main antibodies are mTOR, S6K1, p-mTOR (Ser 2448), p-S6K1 (Thr 389), *β*-actin (Cell signal Technology Company, Boston, USA), BDNF (Affinity, USA), and MBP (Proteintech, USA). The membranes were then incubated with horseradish peroxidase- (HRP-) conjugated secondary antibody. The banding patterns were finally visualized with enhanced chemiluminescence (WP20005, Thermo Fisher Scientific, California, United States), and densitometry was analyzed by VisionWorks software (Eastman Kodak Company, New York, United States).

### 2.7. Cell Viability Assay

Cells were cultured in 96-well plates at a density of 10000 cells per well. After cultured with 5 Mm glucose (C), 100 mM glucose (HG), and 5 mM glucose + 95 mM mannitol group (mannitol) for 24 hours and 48 hours, the cell viability was detected by Cell Counting Kit-8 (CCK-8) (Dojindo Laboratories, Japan).

### 2.8. Statistical Analysis

Data are shown as the mean ± standard deviation (SD), analyzed with GraphPad Prism 7.0 software. All data passed tests for normality and homogeneity of variance. Student's *t*-test and one-way analysis of variance (ANOVA) were used for statistical analyses. Multiple comparisons between groups were analyzed using Tukey's multiple comparisons test. Statistical significance was accepted when *P* < 0.05.

## 3. Results

### 3.1. Establishment of Diabetic Mouse Model, Evaluation of Donor Islets Activity, and Islet Transplantation Effect

**Figure 1:**
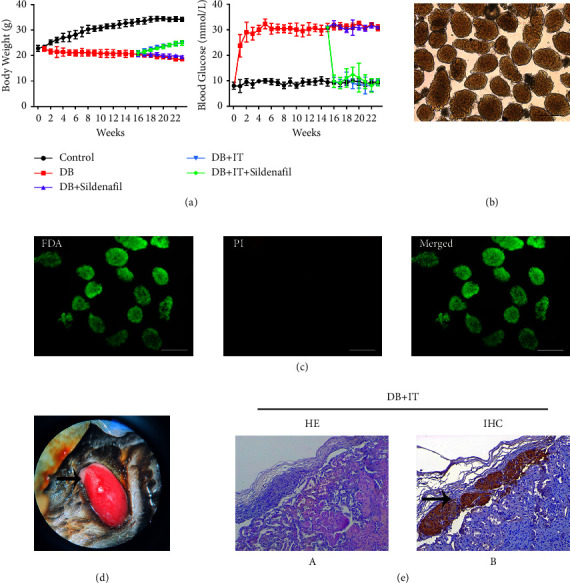
Establishment of the diabetes model, evaluation of donor islet activity, and effect of islet transplantation. (a) The body weight and blood glucose of mice in each group during the whole experiment. (b) Islets extracted from the pancreas of healthy donor mice. (c) Islet transplantation under the renal capsule (Scale = 200 *μ*m). (d) FDA-PI staining used to detect the activity of isolated islets during culture (scale = 200 *μ*m). (e) The results of HE staining (A) and insulin antibody immunohistochemical staining (B) of the kidney after islet transplantation in mice (immunohistochemical staining: ×200).

Following the administration of streptozotocin (STZ) injection for five consecutive days and a normal diet for one week, blood glucose concentrations were determined in C57BL/6 mice by collecting blood from the tail vein. The diabetic mice exhibited a significant increase in blood glucose levels compared with normal mice, and their body weight decreased progressively with the development of diabetes (Figure 1(a)).

Healthy male C57BL/6 mice were anesthetized, and their pancreatic tissues were extracted, digested, and subjected to gradient centrifugation to isolate high-purity islets (Figure 1(b)). After 4–6 hours of culture, some islets were randomly selected for FDA-PI fluorescence staining. Under the fluorescence microscope, the cells with good activity emitted green fluorescence and the cells with weak activity emitted red light. FDA-PI staining results showed that the extracted islet cells maintained good activity, and the proportion of active cells in islets was more than 95% (Figure 1(c)).

Six weeks following subcapsular islet transplantation (Figure 1(d)), the transplanted kidneys were removed for tissue sections. HE staining and immunohistochemical staining revealed that tissues under the renal capsule were capable of normal insulin secretion (Figure 1(e): arrow pointing to the positive site of insulin expression and secretion).

According to the records of body weight and random blood glucose of each group during the experiments, the body weight of diabetic mice began to increase gradually after islet transplantation. While the blood glucose level of the transplantation group exhibited fluctuations, it was significantly distinct from that of diabetic mice without islet transplantation and closely approximated the blood glucose level of normal mice. Administration of sildenafil alone showed no signs of improvement in body weight and blood glucose.

### 3.2. Islet Transplantation Reduces the Pathological Injury of the Sciatic Nerve in Diabetic Mice

**Figure 2:**
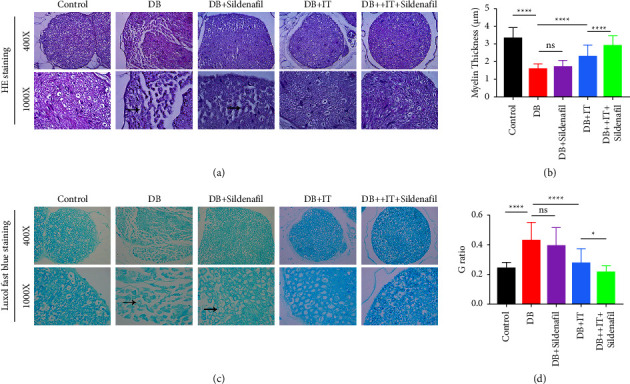
Pathological staining, myelin thickness, and *G* value of sciatic nerve sections in each group. (a) HE staining of sciatic nerve tissue of mice in each group. (b) The thickness of sciatic nerve fiber myelin sheath in each group. (c) Fast blue staining of the myelin sheath of the sciatic nerve of each group (*n* = 3). The arrow points to a thinned and twisted myelin sheath in diabetic mice. (d) *G* value of mice in each group. ^*∗*^*P* < 0.05, ^*∗∗∗∗*^*P* < 0.0001.

Through the results of HE staining of sciatic nerve sections in mice, we can observe the atrophy and distortion of sciatic nerve tissue in diabetic mice. After islet transplantation, most of the sciatic nerve morphology returned to normal (Figure 2(a)). Myelin structure is an important part of nerve fibers. We stained the myelin sheath of the sciatic nerve of mice by fast blue staining and measured the myelin sheath thickness (Figure 2(c)). It was found that the myelin sheath became thinner in the diabetic group (Figure 2(b)). By measuring the diameter of axons and nerve fibers, calculating the ratio (*G* ratio), it was found that the *G* ratio of diabetic mice increased (Figure 2(d)). The *G* ratio is related to the state of nerve fibers, especially the conduction of nerve signals, which further indicates the decrease of the thickness of the myelin sheath and the damage of myelin sheath. After islet transplantation, the injured myelin sheath recovered, the thickness increased, and the *G* ratio decreased.

### 3.3. Islet Transplantation Restored the Expression of Nutritional Factors and Reduced the Expression of Apoptotic Proteins in the Sciatic Nerve of Diabetic Mice

**Figure 3:**
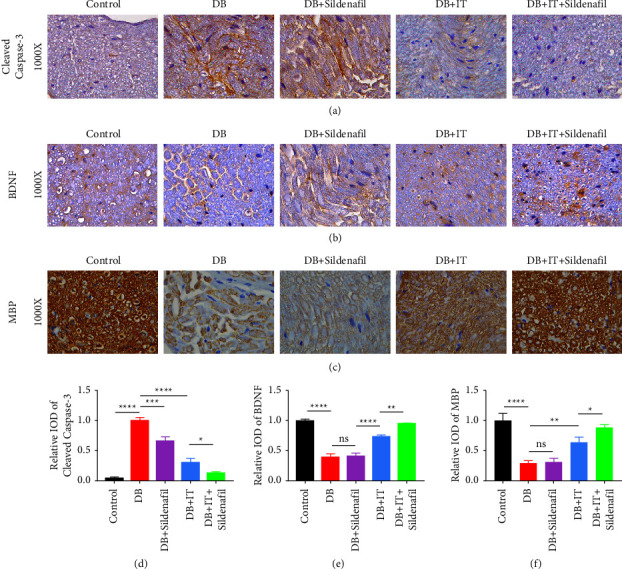
Immunohistochemical staining of sciatic nerve tissue sections of mice in each group. (a–c) IHC analysis was used to detect the expression of cleaved caspase-3, BDNF, and MBP in each group (*n* = 3). (d–f) Quantifications of whose expression in the sciatic nerve was measured by mean integrated optical density (IOD). ^*∗*^*P* < 0.05, ^*∗∗*^*P* < 0.01, ^*∗∗∗*^*P* < 0.001, and ^*∗∗∗∗*^*P* < 0.0001.

The expression of structural and nutritional factors and apoptotic proteins in the sciatic nerve tissue of mice in each group was detected by immunohistochemical staining to further evaluate the state of nerve fibers in each group. According to the semiquantitative analysis of the expression of each protein, we can observe that compared with the normal group, the expression of apoptotic protein cleaved caspase-3 in the sciatic nerve of diabetic mice is significantly increased (Figures 3(a) and 3(d)), while the expression of BNDF (Figures 3(b) and 3(e)) and MBP (Figures 3(c) and 3(f)) protein is decreased, indicating that the nerve fiber myelin sheath is damaged. In the transplantation group, the expression of cleaved caspase-3 decreased, while the expression of BDNF and MBP increased, indicating a restoration of the nerve fiber myelin sheath.

### 3.4. Sildenafil Can Enhance the Therapeutic Effect of Islet Transplantation on Sciatic Nerve Injury in Diabetic Mice

Based on the aforementioned experimental results, the present study elucidates that islet transplantation has the potential to ameliorate sciatic nerve injury in diabetic mice. In addition, our findings indicate that while the diabetic mice treated solely with sildenafil did not exhibit a notable repair effect, the combined treatment of islet transplantation and sildenafil resulted in a more apparent recovery when compared to the group treated only with islet transplantation, as demonstrated by the results in Figures 2 and 3.

### 3.5. Islet Cells Attenuate RSC96 Cell Injury Induced by High Glucose by Upregulating the mTOR/S6K1 Pathway

**Figure 4:**
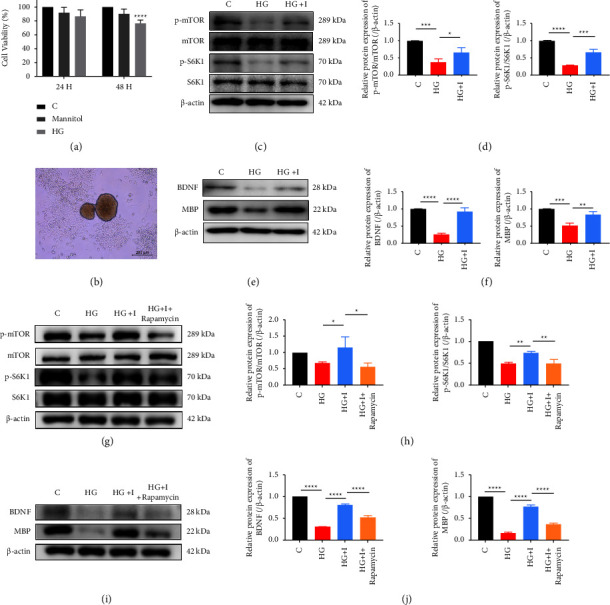
Effects of islets on mTOR/S6K1 phosphorylation and expression of BDNF and MBP proteins in RSC96 cells under high glucose conditions. (a) The results of viability assay (CCK-8) of RSC96 cells cultured in high glucose for 24 and 48 hours. (b) Pancreatic islets were cocultured with RSC96 cells. (c, d) The level and semiquantitative analysis of total protein and phosphorylated protein of mTOR/S6K1 in RSC96 cells cocultured with islets under high glucose condition. (e, f) The level and semiquantitative analysis of BDNF and MBP proteins in RSC96 cells cocultured with islets under high glucose condition. (g, h) The analysis of total protein and phosphorylated protein of mTOR/S6K1 in RSC96 cells cocultured with islets following rapamycin pretreatment under high glucose condition. (i, j) The analysis of BDNF and MBP proteins in RSC96 cells cocultured with islets following rapamycin pretreatment under high glucose condition. ^*∗*^*P* < 0.05, ^*∗∗*^*P* < 0.01, ^*∗∗∗*^*P* < 0.001, and ^*∗∗∗∗*^*P* < 0.0001.

The present study investigated the effect of islet transplantation on RSC96 cells under high glucose condition. Results from the CCK-8 experiment demonstrated a significant decrease in the activity of RSC96 cells cultured in high glucose (100 mmol/L) for 48 hours (Figure 4(a)). At the same time, there was no difference in the activity between the mannitol group and the normal group, excluding the effect of osmotic pressure on cell damage. To further observe the effect of islets on RSC96 cells under high glucose condition, we cocultured the extracted islets with RSC96 cells (Figure 4(b)). Western blotting results showed that the phosphorylation levels of mTOR and S6K1 in RSC96 cells were significantly decreased in the high glucose group, but this phenomenon was reversed in RSC96 cells in the islets coculture group, and the degree of phosphorylation was restored (Figures 4(c) and 4(d)). Similarly, the expression of BNDF and MBP proteins decreased under high glucose and was downregulated after islet coculture (Figures 4(e) and 4(f)).

To further verify the protective effect of islet cells on this pathway, we used rapamycin (0.1 *μ*mol/L), a potent inhibitor of the mTOR pathway. The addition of rapamycin to the coculture system resulted in the attenuation of islets on mTOR/S6K1 pathway functionality in RSC96 cells (Figures 4(g) and 4(h)). In addition, the level expression of BDNF and MBP decreased at the same time (Figures 4(i) and 4(j)). Collectively, our findings suggest that islet transplantation can protect RSC96 cells against high glucose-induced damage by restoring the mTOR/S6K1 pathway and upregulating the expression of BNDF and MBP proteins.

### 3.6. Sildenafil Can Enhance the Therapeutic Effect of Islet Cells against RSC96 Cell Damage Induced by High Glucose

**Figure 5:**
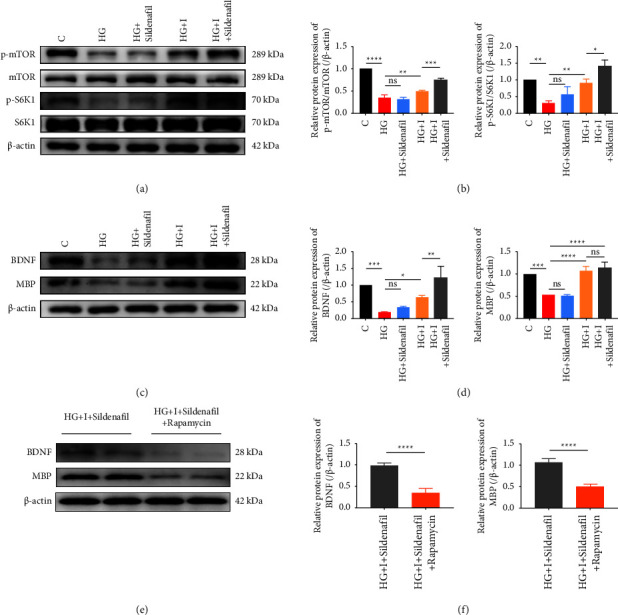
Effects of sildenafil combined with islets on mTOR/S6K1 phosphorylation and expression of BDNF and MBP proteins in RSC96 cells under high glucose conditions. (a, b) The level and semiquantitative analysis of mTOR/S6K1 total protein and phosphorylated protein in RSC96 cells cocultured with sildenafil and islets under high glucose condition. (c, d) The level and semiquantitative analysis of BDNF and MBP proteins in RSC96 cells cocultured with sildenafil and islets under the condition of high glucose. (e, f) The analysis of BDNF and MBP proteins in RSC96 cells cocultured with sildenafil and islets following rapamycin pretreatment under high glucose condition. ^*∗*^*P* < 0.05, ^*∗∗*^*P* < 0.01, ^*∗∗∗*^*P* < 0.001, and ^*∗∗∗∗*^*P* < 0.0001.

In further experiments, sildenafil (0.1 *μ*mol/L) was added to the system in which islets were cocultured with RSC96 cells under high glucose. Similar to the results in animal experiments, we observed that sildenafil alone had limited effect on RSC96 cells. However, upon the addition of sildenafil to the coculture system, a more pronounced mTOR/S6K1 phosphorylation level was observed in RSC96 cells when compared to the coculture group (Figures 5(a) and 5(b)), and the expression level of BDNF was also higher at the same condition (Figures 5(c) and 5(d)). Moreover, we selected rapamycin to further verify the protective effect of sildenafil on islet cells by this pathway. The results of WB showed that after the addition of rapamycin to the coculture system which was added sildenafil, the expression of BDNF and MBP decreased (Figures 5(e) and 5(f)).

## 4. Discussion

The present investigation aimed to explore the role of sildenafil combined with islet transplantation in repairing Schwann cell injury in diabetic peripheral neuropathy. First of all, we established a model of diabetic mice and administered different intervention treatments to each group after stabilizing their diabetic state. Healthy adult mice were utilized as islet donors to be transplanted into the model mice. All diabetic mice who underwent islet transplantation exhibited a normalization of blood glucose levels and gradual weight gain, which was significantly different from diabetic mice who did not receive transplantation. In the further pathological staining observation, the abnormal sciatic nerve tissue, the thinning and twisting of myelin sheath, and the increase of *G* ratio could be seen in the diabetic group. These phenomena were improved to some extent in the transplantation group, and immunohistochemical staining revealed that the expression of BDNF and MBP proteins in the sciatic nerve of the transplantation group returned nearly to normal, and the apoptotic protein cleaved caspase-3 decreased significantly. Furthermore, although the mice treated with sildenafil alone demonstrated normal performance in all aspects, the repair effect of nerve tissue injury was more apparent in the group that underwent transplantation combined with sildenafil treatment. In addition, we grouped RSC96 cells in the same manner of intervention and observed a significant decrease in the protein phosphorylation level of the mTOR/S6K1 pathway in high glucose (100 mmol/L) cultures, accompanied by downregulation of BNDF and MBP protein expression, which was consistent with previous studies. Upon further detection of protein expression in the coculture group, the phosphorylation level of the mTOR/S6K1 pathway upregulated and the expression of BDNF and MBP increased. Similar to the outcomes of animal experiments, we noticed that the restoration of abnormal expression caused by high glucose was more pronounced in cells cocultured with sildenafil and islets.

DPN is one of the earliest and most common complications in patients with type 1 and type 2 diabetes, which is estimated to occur in about half of patients with diabetes [26]. The onset of DPN is relatively hidden, and the progress of the disease is slow. A small number of patients will feel nerve pain, but the vast majority of patients have no obvious symptoms until the late stage of the disease, such as diabetic foot ulcers, gangrene, and other symptoms [27]. Advanced DPN has a great impact on the life treatment of patients with diabetes and brings a heavy burden on patients and their families physically and psychologically.

Schwann cells form a myelin sheath structure and secrete neurotrophic factors to maintain the structure and function of peripheral nerves. Schwann cells also play an important role in the pathogenesis of DPN [28]. The morphological study of diabetic nerves found that there were periodic demyelination and myelin regeneration in the sural nerve, which suggested that Schwann cells were abnormal [29, 30]. Notably, Schwann cells secrete various neurotrophic factors, such as nerve growth factor (NGF), brain-derived neurotrophic factor (BDNF), and neurotrophic factor 3 (NT-3), which are essential for nerve survival and function. Clinical studies have found that serum levels of NGF and BDNF are significantly decreased in patients with DPN [31], and similar results were observed in the culture of primary Schwann cells of type I and type II diabetic mice and immortal rat Schwann cells under high glucose conditions [32–34]. At the same time, a variety of signaling pathways in Schwann cells cultured with high glucose were abnormal, and finally, high glucose would induce the apoptosis of Schwann cells [35]. In Schwann cells treated with high glucose, the protein levels of p-mTOR and Bcl2 decreased, and the expression of these proteins returned to normal after treatment, and the apoptosis of Schwann cells also decreased [8, 35–37]. Therefore, through the regulation of these signal pathways and the supplement of nutritional factors, the damage caused by DPN may have the potential to be repaired.

In our study, we found that islets can restore the abnormal signal of mTOR/S6K1 pathway in Schwann cells, that is, restore the level of phosphorylation, activate the pathway, and increase the expression of BDNF, which fully shows the protective effect of islet cells on Schwann cells and confirmed by animal experiments. Previous experiments have found that the axons of the sciatic nerve of STZ-induced diabetic rats are atrophied and irregular, and the boundary of myelin sheath is unclear [36]. Similar phenomena have been observed in the sciatic nerve of diabetic mice. What is exciting is that the islet transplant reversed these damages. Consequently, our results demonstrate that islet transplantation can counteract the pathological changes in the sciatic nerve of diabetic mice.

Islet transplantation is a promising treatment for patients who have suffered from type 1 diabetes for more than 5 years and developed severe hypoglycemia unawareness [38]. It can maintain the blood glucose level of patients in a stable and reliable way and prevent hypoglycemia and large fluctuations of blood glucose more effectively than exogenous insulin injection, and the risk is lower than that of whole pancreas transplantation [10]. However, several obstacles impede its widespread clinical application, including a shortage of qualified islet donors and the technical challenge of islet purification [39]. At the same time, as a graft, the maintenance of long-term function of transplanted islets will also be affected by many factors, including immune rejection, limited proliferation of transplanted cells, necrosis, and apoptosis [10]. Therefore, it is necessary to develop strategies to improve the activity of transplanted islets or reduce their loss during transplantation.

Sildenafil, a vasodilator, was initially developed for the treatment of neonatal pulmonary hypertension, but now, it is mostly mentioned because of its amazing effect in the treatment of erectile dysfunction in men. Beyond this, sildenafil has demonstrated its versatility as a potential therapeutic agent in numerous fields. Members of the PDE-5i drug family, including sildenafil, are currently being investigated as a new direction for cancer treatment [40]. In the realm of transplantation, studies have revealed that sildenafil can promote the recovery of myocardial function after ischemia associated with heart transplantation [41] and aid in the success of lung, liver, and kidney transplantation [42–44]. In addition, an *in vitro* study has demonstrated that sildenafil citrate can affect the immune system by reducing the production of TNF-*α* by T lymphocytes [45]. It shows that sildenafil may have a certain immunosuppressive effect. This coincides with the research direction of the protection of transplanted islet cells by TNF-*α* inhibitors [46].

Therefore, we introduced sildenafil into the experiment of islet transplantation, hoping to explore whether sildenafil can play a positive role in islet transplantation. The results were satisfactory and we observed that with the help of sildenafil, whether cocultured in vitro or transplanted *in vivo*, the protective effect of islets was magnified to a certain extent. This finding provides a new direction of thinking for maintaining the long-term and stable activity of islet cells. If those effects can be further confirmed, sildenafil can reduce the dosage of donor islets or the loss during inhibition to a certain extent in the case of lack of donor islet resources and provide the possibility of islet transplantation for more patients with diabetes and its complications. Further confirmation of these effects will be instrumental in advancing the development of new and effective therapies for this patient population.

The present study, however, has several limitations that require further investigation. In the next work, we hope to explore the possibility of reducing the required amount of islet transplantation with sildenafil. We propose the establishment of a reduced transplantation group combined with sildenafil, which can be compared with the normal transplantation group. If there are no significant differences between the two groups, we can confirm our hypothesis. In addition, for the study of DPN, because of the limitations of experimental conditions, it is a pity that we do not have relevant behavioral records in animal experiments, which we hope to be supplemented and improved in the following research.

## 5. Conclusion

Our experiments confirmed that islet transplantation significantly improved blood glucose and body weight of diabetic mice, restored the morphological structure of the myelin sheath of the sciatic nerve, and upregulated the expression of BDNF and MBP in the tissue. By regulating the mTOR/S6K1 pathway, islet cells can protect Schwann cells cultured in high glucose. At the same time, in animal experiments and cell experiments, it was observed that the effect of islet cells was increased under sildenafil combined therapy. This provides a new idea for individualized efficacy evaluation and targeted therapy in patients with diabetes mellitus and its complications.

## Data Availability

The data used to support the findings of this study are available from the corresponding authors upon request.

## Abbreviations

DPN: Diabetic peripheral neuropathy
BDNF: Brain-derived neurotrophic factor
MBP: Myelin basic protein
PDE5 inhibitor: Phosphodiesterase-5 inhibitor
mTOR: Mammalian target of rapamycin
S6K1: Ribosomal protein S6 kinase 1
STZ: Streptozotocin.
